# A survey of vocal health in church choir singers

**DOI:** 10.1007/s00405-021-06770-0

**Published:** 2021-04-10

**Authors:** Vasudha Sharma, Srikanth Nayak, Usha Devadas

**Affiliations:** 1 Department of Speech and Hearing, Manipal College of Health Professions, Manipal Academy of Higher Education, Manipal, Karnataka 576104 India; 2 Department of Audiology and Speech Language Pathology, Yenepoya University (Deemed to be University), Mangalore, Karnataka 575018 India

**Keywords:** Choir singers, Singers, Voice problem, Vocal symptoms, Risk factors, Vocal health

## Abstract

**Purpose:**

Choir singing is an important tradition of Christian worship across India. However, vocal health issues related to the church choir singers are less addressed in the literature. Hence, this study aimed to investigate the prevalence of vocal symptoms, identify the variables associated with increased risk of voice problems and knowledge of factors influencing vocal health in church choir singers.

**Method:**

One hundred and forty-eight church choir singers (61 males and 85 females) between the age range of 18 and 70 years participated in the study. They completed a self-reported questionnaire addressing demographic and singing-related details, vocal symptoms, variables associated with increased risk reporting voice problems and knowledge about factors influencing vocal health.

**Result:**

Eighty-four percent of the choir singers reported two or more vocal symptoms sometimes or more frequently while or after singing. More than half of the church choir singers had experienced vocal symptoms such as accessing notes in the upper range, loss of vocal endurance, pitch breaks, hoarseness, dryness in the throat, and discomfort in the throat. Among the different variables, systemic hydration found to have a significant association with reporting of voice problems in church choir singers. The overall knowledge regarding the factors influencing vocal health was found to be limited among the choir singers.

**Conclusion:**

Choir singers like other professional singers experienced a higher prevalence of vocal symptoms during or after singing and exhibited limited knowledge about factors that negatively influence vocal health. Hence, there is a need to look into these singer’s vocal requirements, who usually go unnoticed.

## Introduction

Choral music popularized by western missionaries for spreading of Christian gospel outside European countries [1] and it is practised in religious, school and community settings. In choir singing, an organized company of singers perform music together in a chorus of voices, often with harmony parts designed to make the choral songs sound full. In choir singing, one person may sing the music’s melody (i.e. Soprano) and the other singers sing in harmony (alto, tenor and bass), which are complementary lines of music that make up the chords. Amateur and trained professional singers of different age groups participate in choir singing in church. Choir singing requires the singers to contribute to the choral sound without dominating it and responding to the artistic demands set by the choir in charge/leader [2]. The technical singing demands of choral singing differs from other forms of singing in terms of spectral characteristics, loudness level and pitch. As choral singers need to blend their voice with other singers in the group, constant adjustments in loudness, pitch, and timbre are required to match their voice with other singer’s voices [3]. However, in some instances, choir conductors have relatively little information regarding tone production and ascertain most of their knowledge through experimentation [4]. This could lead to problems in vocal training and vocal development in the long run.

Singing demands good coordination between respiratory and laryngeal systems. Hence, singers are considered elite vocal performers with increased vocal demand [5] and at greater risk of developing voice problems [6]. Several studies in the literature have identified a higher prevalence of voice problems in different professionally trained singers [7–18]. However, vocal health issues in choral singers are less addressed in the literature. A study conducted in Australia reported poor vocal health care knowledge among Contemporary worship singers [19]. Another study reported suboptimal choral singing behaviors (singing outside comfortable pitch range, singing too loudly, singing too softly) having a positive correlation (*r* = 0.34, *p* < 0.0001) with experiencing vocal fatigue in choral singers [3]. Neto and Meyer [20] investigated the vocal health and awareness of vocal hygiene principles among 614 contemporary commercial Christian singers. The study results indicated symptoms such as vocal fatigue (34.7%), tickling and choking sensation in the throat (24.3%), loss of upper range (28%) and complete loss of voice (4%) were prevalent among these group of singers. Further, the study revealed that more than half of the participants were amateur singers with poor knowledge of vocal hygiene practices. A recent study conducted in Sao Paulo among 526 amateur choristers analyzed the relationship between vocal risk and voice signs and symptoms, specific singing handicap and generalized anxiety. The choristers who were identified with vocal risk (scored more than the cut-off score on VoiSS) reported higher singing voice handicap score on the Modern Singing Handicap Index (MSHI) and mild anxiety on Generalized Anxiety Disorder 7-item (GAD-7) scale [21]. A study conducted in Brazil showed an inclination to dysphonia in university choir singers. Throat pain, throat clearing, hoarseness and cough were the most common symptoms experienced by these choirs [22]. Tepe et al. [9] conducted a study on vocal health in 129 young choir singers and reported 56% of the singers had vocal difficulty, of which 43% reported hoarseness in voice and further one-third of the total singers had an incidence of straining and over-singing. Ravall and Simberg [23] investigated the prevalence of voice problem and vocal health and awareness among 315 choir singers. The study results indicated the 21% of participants experienced two or more frequently occurring vocal symptoms and 50% of the subjects had limited voice care knowledge.

Like in other countries, choir singing is an important tradition of Christian worship across India. Christian worship in India is highly dependent on the sect of Christianity that one belongs to (Roman Catholic and Protestants). Roman Catholics celebrate mass or the Holy Eucharist wherein the hymn singing and choral repertoire is followed, whereas Protestants follow the praise team approach. In both scenarios, the vocal demands follow the standards set by western classical music, i.e., musical instruments (electronic and acoustic) together with vocal harmonies. An unpublished pilot study conducted on 72 choral singers in Mizoram reported poor vocal hygiene practices [24]. The study reported that church choir singers are generally “sing by ear” trainers where the singers appear to have little experience in reading the musical score at sight and they mostly learn their musical parts (pitch, intervals, melody, chords, rhythms and other fundamental music elements) by listening to them and do not receive any formal classical training. The choir practice frequency ranges from 2 to 3 times per week (around 2 h of practice/session) and some singers may find it difficult to perform specific repertoire in the voice parts assigned if their vocal range does not fit well within the specified singing range. However, voice problems experienced by the Indian church choir singers are less reported in the literature. The purpose of this self-reported survey was to investigate the prevalence of vocal symptoms, identify the variables associated with increased risk of voice problems and knowledge of factors influencing vocal health in church choir singers.

## Methods

### Participants and data collection

The present cross-sectional study was conducted in the Udupi district of Karnataka state, India. After obtaining approval from the institutional research committee, the first author approached 12 church choir leaders and explained the study’s purpose. By obtaining permission from church choir leaders, the first author contacted around 150 choir singers during their practice sessions in different churches and was explained the purpose of the study. The study included church choir singers who were 18 years of age and above with a minimum of 2 years of experience in church choir singing and practised singing for a minimum duration of 2 hours/week. The participants were asked to complete a questionnaire and an informed consent form.

### Survey instrument

For this study, a self-reported questionnaire addressing vocal health and habits in church choir singers was developed taking inputs from similar studies in the literature related to singers [9, 25]. For the content validation, the questionnaire was distributed among five experienced speech language pathologists (SLPs) and were asked to give their suggestions and comments on the content of the questionnaire. After incorporating the suggestions of SLPs, the questionnaire was distributed to five choir singers for the familiarity assessment. They were asked to give their comments and suggestions concerning the familiarity of the terminologies used in the questionnaire, relevance, appropriateness of the questions, and identifying any missing information. Suggestions from the choir singers were included in the questionnaire and distributed to five choir singers once again. As the respondents reported no further ambiguity or difficulty, the questionnaire was accepted for use in this study. The questionnaire included questions on the following domains: demographic and choir singing-related details, vocal symptoms, lifestyle, phonotraumatic and health-related factors, knowledge of factors influencing vocal health (refer Appendix A).

### Statistical analysis

Statistical package for social sciences 20.0 (SPSS, IBM Corp. Released 2011. IBM SPSS Statistics for Windows, Version 20.0. Armonk, NY: IBM Corp.) was used for the statistical analysis. Mean and the standard deviation was used to summarize continuous variables and the percentage was used to describe the categorical variables. The prevalence of vocal symptoms was calculated in percentage. Pearson’s Chi-square test (with 95% confidence interval) was used to compare the differences in the demographic characteristics, voice symptoms, health-related factors, and lifestyle-related and phonotraumatic factors and knowledge related to factors influencing vocal health between choir singers with voice problem (VP) and no voice problem (NVP).

## Results

A response rate of 98.66% was obtained with returning of 148 completed questionnaires. After excluding two incomplete questionnaires, the study results were analyzed from 146 questionnaires which included responses from 61 (41.78%) males and 85 (58.21%) females choir singers with a mean age of 26.77 ± 12.07 years (range between 18 and 70 years). As shown in Table 1, more than 50% of the choir singers were between the age range of 18 and 30 years with singing experience between 2 and 20 years. Around 69% (*n* = 101) of the singers not received any formal training and 60% (*n* = 88) of them do not warm up their voice prior to the singing performance. About 26.7% (*n* = 39) of the singers reported they need to use their voice in their profession other than choir singing.

**Table 1:** Demographic characteristics and choir singing practice details of the VP and NVP Groups

Demographic characteristics	VP (*n* = 129)	NVP (*n* = 17)	*χ*^2^	*df*	*p* value
	% (*n*)	% (*n*)			
Age					
18–30 years	79.8 (103)	76.4 (13)	0.105	1	0.746
> 30 years	20.2 (26)	23.6 (4)			
Gender					
Male	39.53 (51)	58.82 (10)	2.297	1	0.130
Female	60.46 (78)	41.18 (7)			
Years of choir singing experience					
0–10 years	49.61 (64)	29.41 (5)	5.739	4	0.220
11–20 years	34.88 (45)	47.05 (8)			
21–30 years	7.75 (10)	11.76 (2)			
31–40 years	4.65 (6)	0 (0)			
41–50 years	3.10 (4)	11.76 (2)			
Musical training for choir singing					
Not trained	69.77 (90)	64.70 (11)	0.180	1	0.671
Trained	30.23 (39)	35.30 (6)			
Vocal warm-up exercise to improve voice					
Perform no exercise	62.01 (80)	41.18 (7)	2.709	1	0.100
Perform exercise	37.98 (49)	58.82 (10)			
No. of hours of practice and singing per week					
≤ 3 h	73.6 (95)	70.6 (12)	0.789	1	0.072
> 3 h	26.4 (34)	29.4 (5)			
Profession/job requires voice use					
No	74.42 (96)	64.70 (11)	0.724	1	0.395
Yes	25.58 (33)	35.30 (6)			

Data are percentage (number) of choir singers unless otherwise specified. Percentages were calculated with the number of respondents

*p* values were derived from the chi-square test

### Prevalence of vocal symptoms experienced by choir singers

In this study, to measure the prevalence of vocal symptoms experienced by choir singers, they were asked to indicate whether they experienced any of the vocal symptoms while or after singing listed in the questionnaire using a five-point Likert scale (never, almost never, sometimes, almost always, always). For the purpose of analysis, singer’s rating a symptom sometimes or more frequently was considered as the presence of a vocal symptom and their rating of a symptom ‘almost never’ or ‘never’ was considered as the absence of that vocal symptom. As shown in Table 2, the vocal symptoms such as difficulty hitting or singing higher notes, hoarseness especially in higher pitch range, discomfort in the throat, pitch breaks or momentary loss of voice, dryness in the throat were the most prevalent symptoms experienced by choir singers during or after their singing. Further, in this study, singers who reported two or more symptoms, sometimes or more frequently while singing were classified as singers with ‘voice problem’ (VP) and the rest were classified as ‘no voice problem’ (NVP). Based on this classification, 88.4% (*n* = 129) of choir singers were classified as singers with VP with a mean age of 26.26 ± 11.69 years, and 11.6% (*n* = 17) were classified as NVP with a mean age of 30.65 ± 14.50 years.

**Table 2.** The prevalence of individual vocal symptoms reported by the choir singers while, or after singing

Vocal symptom	Prevalence % (*n*)
Difficulty in accessing notes in the upper range	75.3 (110)
Difficulty making smooth register transitions	58.2 (85)
Loss of vocal endurance and flexibility	67.1 (98)
Hoarseness, especially in higher pitch range	69.9 (102)
Pitch breaks or momentary loss of voice	61 (89)
Discomfort in the throat	67.8 (99)
Pain in the throat	50 (73)
Dryness in throat	63 (92)
Tightness in the throat	28.8 (42)
Pain in the throat	50 (73)

### Comparison of demographic, singing, vocal symptom, lifestyle, phonotraumatic, lifestyle, health-related factors and knowledge of factors influencing vocal health between choir singers with (VP) and without reporting voice problems (NVP)

Using Pearson’s Chi-square test, this study compared the demographic, singing, vocal symptoms, lifestyle, phonotraumatic, and health-related factors between choir singers reporting VPs (VP) and those who did not report VPs (NVP). As shown in Table 1, even though Pearson’s Chi-square test revealed statistically no significant difference between the VP and NVP group with respect to demographic and singing-related factors relatively higher number of female singers reported having voice problem compared to male singers. Whereas, choir singers in the VP group reported all the vocal symptoms at a significantly higher rate (*p* < 0.05) than choir singers in the NVP group (Table 3). Further, choir singer’s comparison of lifestyle, phonotraumatic and health-related factors is shown in Table 4 and Table 5, respectively. As shown in Table 4, the prevalence of reporting voice problem was significantly higher in choir singers who consumed less than seven glasses of water/day. Even though there was no statistically significant difference observed in reporting phonotraumatic behaviors between two groups of choir singers, a marginally higher percentage of choir singers with VP reported they run out of breath while singing compared to choir singers in the NVP group which could be inefficient singing technique rather than respiratory pathology. No significant difference was found between VP and NVP groups regarding general health-related factors (Table 5). However, choir singers with VP experienced a higher rate of upper respiratory tract infection, visited a doctor for their voice problem, taken medication, received voice rest and voice therapy than singers reporting NVP.

**Table 3:** Prevalence of individual vocal symptoms reported by VP and NVP group of choir singers while, or after singing

Symptom	VP (*n* = 129)	NVP (*n* = 17)	*χ*^2^	*df*	*p* value
	% (*n*)	% (*n*)			
Accessing notes in the upper range					
No	14.7 (19)	100 (17)	58.790	1	< 0.001
Yes	85.3 (110)	0 (0)			
Difficulty making smooth register transitions					
No	34.1 (44)	100 (17)	26.810	1	< 0.001
Yes	65.9 (85)	0 (0)			
Loss of vocal endurance and flexibility					
No	26.4 (34)	82.4 (14)	21.342	1	< 0.001
Yes	73.6 (95)	17.6 (3)			
Hoarseness, especially in the higher pitch range					
No	20.9 (27)	100 (17)	44.603	1	< 0.001
Yes	79.1 (102)	0 (0)			
Pitch breaks or momentary loss of voice					
No	33.3 (43)	82.4 (14)	15.166	1	< 0.001
Yes	66.7 (86)	17.6 (3)			
Discomfort in the throat					
No	24 (31)	94.1 (16)	33.801	1	< 0.001
Yes	76 (98)	5.9 (1)			
Pain in the throat					
No	43.4 (56)	100 (17)	19.240	1	< 0.001
Yes	56.6 (73)	0 (0)			
Dryness in the throat					
No	34.1 (44)	58.8 (10)	3.937	1	0.047
Yes	65.9 (85)	41.2 (7)			
Vocal fatigue					
No	51.2 (66)	76.5 (13)	3.874	1	0.049
Yes	48.8 (63)	23.5 (4)			
Tightness in the throat					
No	68.2 (88)	94.1 (16)	4.917	1	0.027
Yes	31.8 (41)	5.9 (1)			

Data are percentage (number) of choir singers unless otherwise specified. Percentages were calculated with the number of respondents

*p* values were derived from the chi-square test

Values indicate statistical significance (< 0.005)

**Table 4:** Lifestyle and phonotraumatic factors of the VP and NVP groups of choir singers

Lifestyle factors and phonotraumatic behaviors	VP	NVP	*χ*^2^	*df*	*p* value
	(*n* = 129)	(*n* = 17)			
	% (*n*)	% (*n*)			
Lifestyle habits					
Smoking					
No	87.6 (113)	88.2 (15)	0.006	1	0.940
Yes	12.4 (16)	11.8 (2)			
Alcohol					
No	59.7 (77)	64.7 (11)	0.158	1	0.691
Yes	40.3 (52)	35.3 (6)			
Eating late at night					
No	56.6 (73)	52.9 (9)	0.081	1	0.776
Yes	43.4 (56)	47.1 (8)			
Eat spicy/oily food					
No	19.4 (25)	35.3 (6)	2.275	1	0.132
Yes	80.6 (104)	64.7 (11)			
Eat ice cream/chocolates often					
No	33.3 (43)	41.2 (7)	0.410	1	0.522
Yes	66.7 (86)	58.8 (10)			
Drink 7–8 glasses of water/day					
No	50.4 (65)	0 (0)	15.440	1	< 0.001
Yes	49.6 (64)	100 (17)			
Vocal abusive behaviors					
Speak with a loud voice					
No	51.2 (66)	58.8 (10)	0.353	1	0.552
Yes	48.8 (63)	41.2 (7)			
Usually speak fast					
No	51.2 (66)	64.7 (11)	1.105	1	0.293
Yes	48.8 (63)	35.3 (6)			
Run out of breath while talking					
No	85.3 (110)	100 (17)	2.878	1	0.090
Yes	14.7 (19)	0 (0)			
Excessive talking					
No	50.4 (65)	52.9 (9)	0.039	1	0.843
Yes	49.6 (64)	47.1 (8)			

Data are percentage (number) of choir singers unless otherwise specified. Percentages were calculated with the number of respondents

*p* values were derived from the chi-square test

Values indicate statistical significance (< 0.005)

**Table 5:** General health and vocal health factors in VP and NVP groups of choir singers

General and vocal health factors	VP	NVP	*χ*^2^	*df*	*p* value
	(*n* = 129)	(*n* = 17)			
	% (*n*)	% (*n*)			
General health					
Frequent URTI episodes					
No	77.5 (100)	88.2 (15)	1.031	1	0.310
Yes	22.5 (29)	11.8 (2)			
Experience frequent heartburn					
No	83.7 (108)	88.2 (15)	0.231	1	0.631
Yes	16.3 (21)	11.8 (2)			
Vocal health care					
Been to a doctor for voice problem					
No	90.7 (117)	94.1 (16)	0.217	1	0.642
Yes	9.3 (12)	5.9 (1)			
Taken medication for voice problem					
No	93 (123)	100 (17)	1.264	1	0.261
Yes	7 (9)	0 (0)			
Been put on voice rest for voice problem					
No	87.6 (113)	100 (17)	2.368	1	0.124
Yes	12.4 (16)	0 (0)			
Received voice therapy for voice problem					
No	97.7 (126)	100 (17)	0.404	1	0.525
Yes	2.3 (3)	0 (0)			

Data are percentage (number) of choir singers unless otherwise specified. Percentages were calculated with the number of respondents. *p* values were derived from the chi-square test

### Knowledge about factors influencing vocal health

From the list of the different risk factors, 82% (*n* = 120) of the choir singers considered yelling and shouting as the risk factor for developing the voice problem. More than 60% of the choir singers answered excessive speaking with a loud voice (63%; *n* = 92), talking during throat infection (68.4%; *n* = 100), talking in the presence of loud background noise (67%; *n* = 98) and smoking (69%; *n* = 101) as risk factors for developing voice problems. However, relatively a smaller number of them considered emotional stress (42.4%; *n* = 62), excessive throat clearing (38.3%; *n* = 56), acid reflux (43%; *n* = 63), nasal allergies (56.8%; *n* = 83) and whispering (25.3%; *n* = 37) as risk factors for developing voice problems. Further, when the knowledge about risk factors causing voice problems compared between VP and NVP group of choir singers using Pearson’s Chi-square test (Table 6) significantly higher number of choir singers in the NVP group identified yelling and shouting and allergies as risk factors for the development of voice problems than VP group.

**Table 6:** Knowledge about factors influencing vocal health

Factors	VP	NVP	*χ*^2^	*df*	*p* value
	(*n* = 129)	(*n* = 17)			
	% (*n*)	% (*n*)			
Emotional stress					
Yes	45 (58)	35.3 (6)	0.570	1	0.450
No	55 (71)	64.7 (11)			
Yelling and shouting					
Yes	84.5 (109)	64.7 (11)	4.019	1	0.045
No	15.5 (20)	35.3 (6)			
Excessive Speaking with loud voice					
Yes	62.8 (81)	64.7 (11)	0.024	1	0.878
No	37.2 (48)	35.3 (6)			
Excessive throat clearing					
Yes	37.2 (48)	47.1 (8)	0.616	1	0.432
No	62.8 (81)	52.9 (9)			
Talking during throat infection					
Yes	68.2 (88)	70.6 (12)	0.039	1	0.843
No	31.8 (41)	29.4 (5)			
Smoking					
Yes	69 (89)	70.6 (12)	0.018	1	0.893
No	31 (40)	29.4 (5)			
Acid reflux indigestion					
Yes	44.2 (57)	35.3 (6)	0.484	1	0.487
No	55.8 (72)	64.7 (11)			
Whispering					
Yes	25.6 (33)	23.5 (4)	0.033	1	0.855
No	74.4 (96)	76.5 (13)			
Allergies					
Yes	60.5 (78)	29.4 (5)	5.905	1	0.015
No	39.5 (51)	70.6 (12)			
	44.2 (57)	52.9 (9)	0.465	1	0.495
	55.8 (72)	47.1 (8)			

Data are percentage (number) of choir singers unless otherwise specified. Percentages were calculated with the number of respondents

*p* values were derived from the chi-square test

Values indicate statistical significance (< 0.005)

## Discussion

This study aimed to investigate the prevalence of vocal symptoms, identify the variables associated with increased risk of voice problems and knowledge about factors influencing vocal health in church choir singers. In this study, the data collection method (first author directly contacted choir singers and responses collected on the same day) would have resulted in a higher response rate (98.66%). Church choir singers in India are not trained in par with professional singers but are engaged in similar kind of vocal activities, resulting in the risk of developing vocal problems. However, vocal problems in church choir singers are less reported in India.

### Prevalence of self-reported VPs in choir singers

In this study, more than half of the church choir singers had experienced vocal attrition symptoms such as accessing notes in the upper range, loss of vocal endurance/flexibility, pitch breaks (inadequate negotiation of transitions or areas within a passaggio) or momentary loss of voice, hoarseness, dryness in the throat and discomfort in the throat. Further, 88.4% (*n* = 129) of the choir singers reported two or more vocal symptoms sometimes or more frequently while or after singing. This is consistent with the earlier studies that reported a higher prevalence of voice problems in choir singers [2, 9, 16, 21, 23]. Apart from this, most choir singers in this study were younger age group (18–30 years) and 69% (*n* = 101) were not trained professionally. The findings are in consonance with choir singer’s reports in the literature who reported the choir singers are not professionally trained and perform rehearsals without proper knowledge of vocal techniques [2, 9, 16, 21, 23]. Studies reported that young singers do not apply their good singing techniques while speaking, and many engage in extensive and strenuous singing activities without proper guidance or training [25, 26]. These vocal behaviors could put the young singers at more risk for developing vocal attrition symptoms. Hence, the possibilities of vocal abuse and misuse are very high while speaking and practicing choir singing. Compared to younger singers, experienced choir singers may learn the ability to alter their voice in a more controlled and flexible way and learn how to prevent voice problems than young choir singers [27]. Hence, we can assume that majority of the young choir singers in this study may be involved in inappropriate voice use or abuse of voice while singing or speaking and could be the reason for a higher prevalence of voice problems and needs further investigation to confirm our hypothesis. Even though women are reported to experience higher voice problems than men, in this study no significant difference observed between genders in reporting vocal symptoms while or after singing. Similar findings reported in the literature and the authors hypothesized that like female singers, male choir singers are sensitive to vocal difficulties experienced during singing [23]. This could be true for this study as the choir singers were asked to report vocal symptoms experienced during or after singing and both men and women are highly sensitive towards vocal difficulties experienced during singing.

It is generally expected that singers who are trained should experience lesser voice problems than the singers who do not receive any formal training because trained singers gain better knowledge regarding singing techniques, vocal hygiene, and maintaining good vocal health compared to amateur or untrained singers. However, this was not true for the choir singers in this study who reported they received singing training. Out of 45 (30.8%) choir singers who received singing training, 39 (87%) reported they experienced vocal symptoms. Similar results were postulated in the study conducted by Tepe et al. [9] and Yiu and Chan [28], wherein training did not show any positive impact on choir singing voice. However, in this study, we did not inquire what kind of training the choir singers received and from whom they received it. Hence, it needs further investigation to comment on the choir singer’s singing training and reporting of vocal symptoms.

### Variables associated with increased risk reporting voice problems in choir singers

Contrary to our expectations, Pearson’s Chi-square test did not reveal any significant difference between choir singers with VP and NVP for lifestyle, phonotraumatic and health-related characteristics except hydrating habits. In this study, significantly higher (*p* < 0.001) number of choir singers in the VP group reported consuming less than 7–8 glasses of water than choir singers in the NVP group. Mitrano [29] suggested that singers should drink plenty of water (8–10 glasses) daily, and avoid alcohol and smoking to maintain good vocal fold hydration. For a singer retaining proper hydration is critical to vocal fold performance [30]. Well-hydrated vocal folds vibrate with more ease during singing than less hydrated vocal folds. The vocal folds become less lubricated due to inadequate hydration and hence the vocal fold oscillations become more effortful for the singer [31]. Thus, this finding strengthens the notion that retaining proper hydration is critical for singers. Even though acid reflux, alcohol consumption, intake of medicines, and caffeine are confirmed risk factors for voice problems, they did not show association with reporting of voice problems in this study. This finding could be attributed to the fact that relatively fewer choir singers reported their experience with these agents, or in terms of acid reflux, these singers would have failed to recognize the silent discomfort associated with it. Further, even though none of the other variables was found to have a significant association with experiencing vocal symptoms, it is quite possible that they indirectly influenced the outcome through their association with the variable showing significant association.

### Knowledge about factors influencing vocal health among choir singers

Even though church choir singers do not use their voice to earn a livelihood, they effectively utilize their voice for a considerable duration. Hence, this study intended to understand their knowledge regarding factors harmful for maintaining good vocal health. Among the variables listed, a relatively higher percentage of choir singers identified yelling and shouting, speaking with a loud voice, talking during throat infections, talking in the presence of background noise and smoking as the risk factors for the development of voice problems. However, relatively fewer choir singers identified the negative influence of emotional stress, clearing throat, whispering, and acid reflux on the vocal mechanism, which substantiates earlier studies findings [9, 23]. Therefore, these results depict an immense need to educate these choir singers and impart knowledge regarding vocal hygiene, vocal care techniques, protective voice factors, and causative factors, leading to voice problems that will help them improve their singing voice and experience lesser vocal symptoms.

Seeking medical help for voice problems was found to be poor in choir singers. Even though more than 80% of them reported they experienced voice problems, only 9.3% (*n* = 12) of them visited a physician, 7% (*n* = 9) received medication for their voice problem, and 12.4% (*n* = 16) were advised to voice rest. Further, 2.3% (*n* = 3) of them reported that they had received voice therapy. This finding is in consonance with studies in literature [23] where only 5.1% of the choir singers visited ENT specialist or SLP for their voice problems. The possible explanation for this can be a lack of vocal health education and practice of vocal techniques to maintain a healthy singing voice. Other possible reason could be choir singing is a leisure activity and involves group singing. Even those who may have had voice training for solo repertoire may be unsure of what to do to achieve the choral singing effectively. Hence, choir singers may not give importance to their vocal difficulties like other professional singers and get less psychologically taxed or bothered by their vocal health and very few are likely to seek medical help from physicians or voice pathologist. Understanding the impact of voice problems on choir singers' quality of life can give a better insight in this regard.

## Conclusion

Overall, this study’s findings suggest that more than 80% of the choir singers report two or more vocal symptoms more frequently and its impact on their singing. Most choir singers in India are ear trained and do not receive formal singing training, and they have limited knowledge about factors that can negatively influence singing voice. Hence, there is a great need to look into these singer’s vocal requirements, which usually go unnoticed. In future, SLPs should collaborate with choir conductors and singers to understand the type and nature of singing training received by choral singers. The information gathered can be used to enhance their knowledge regarding vocal hygiene and health care factors, create awareness regarding the identification of the vocal problem and its prompt intervention, and impart education to help prevent voice dysfunction experienced by them.

## A survey of vocal health in church choir singers

Kindly read the following questions and indicate your choice as per the requirement of the question.

### II. Have you experienced any of the following voice problems often, while, or after SINGING (tick the appropriate option for each symptom given below)

**Table:**

Difficulty hitting or singing higher notes	Never	Almost never	Sometimes	Almost always	Always
Difficulty making smooth register transitions	Never	Almost never	Sometimes	Almost always	Always
Loss of vocal endurance and flexibility, especially after singing for more than an hour	Never	Almost never	Sometimes	Almost always	Always
Hoarseness or breathiness, especially in the higher pitch range	Never	Almost never	Sometimes	Almost always	Always
Pitch breaks or momentary loss of voice or other sudden changes in the voice, especially after singing for more than an hour	Never	Almost never	Sometimes	Almost always	Always
Discomfort in the throat, especially after singing for more than an hour	Never	Almost never	Sometimes	Almost always	Always
Pain in the throat, especially after singing for more than an hour	Never	Almost never	Sometimes	Almost always	Always
Dryness in the throat	Never	Almost never	Sometimes	Almost always	Always
Voice fatigue (voice tires easily when talking)	Never	Almost never	Sometimes	Almost always	Always
Tightness in the throat	Never	Almost never	Sometimes	Almost always	Always

### III. Please indicate your experiences with the following (tick YES or NO)

**Table:**

Do you smoke?	Yes	No
Do you consume alcohol?	Yes	No
Do you eat late at night?	Yes	No
Do you eat spicy/oily food?	Yes	No
Do you eat ice cream/chocolates often?	Yes	No
Do you drink at least 7–8 glasses of water a day?	Yes	No
Do you usually speak with a loud voice?	Yes	No
Do you usually speak fast?	Yes	No
While speaking, do you usually "run out of air" or lose your voice at the end of a sentence?	Yes	No
Do you usually find yourself doing most of the talking when conversing with a friend or in a social gathering?	Yes	No
Do you experience frequent upper respiratory tract infection?	Yes	No
Do you experience frequent acid reflux/heart burn?	Yes	No
Been to a doctor because of voice problems?	Yes	No
Taken any medication for voice problems?	Yes	No
Been put on voice rest for voice problem?	Yes	No
Received speech/voice therapy for voice problems?	Yes	No

### VI. Indicate, in your opinion which of the following activities, substances, and environments are harmful to the vocal mechanism

**Table:**

Emotional stress	Yes	No
Yelling and shouting	Yes	No
Excessive speaking with loud voice	Yes	No
Excessive throat clearing	Yes	No
Talking during a throat infection	Yes	No
Smoking	Yes	No
Acid reflux/heart burn	Yes	No
Nasal allergies/Upper respiratory tract infection	Yes	No
Whispering	Yes	No
Talking in the presence of loud background noise	Yes	No

Thank you! Your participation is really appreciated.
